# LaZeTrack: An open‐source object detection algorithm to detect and track 2‐day‐old zebrafish motor touch responses

**DOI:** 10.1002/qub2.70043

**Published:** 2026-06-01

**Authors:** Adrien Lacroix, Gary Alan Barclay Armstrong

**Affiliations:** ^1^ Department of Neurology and Neurosurgery McGill University Montreal Quebec Canada

**Keywords:** automated tracking, machine learning, zebrafish behavioral analysis, zebrafish motor function

## Abstract

Zebrafish are a model organism used for the study of vertebrate development, disease, and drug discovery. Two‐day old zebrafish exhibit burst swimming behavior that can be elicited by a light touch to the tail. Motor touch responses are frequently video‐recorded and later analyzed by hand. Despite this being a simple behavioral assay, methods for robustly analyzing these videos in a reproducible and time‐efficient manner rely on manual tracking, which is prone to experimenter bias and error. Here we present LaZeTrack, a machine learning‐based program, which employs Ultralytics’ YOLOv8 nano (YOLOv8n) object detection algorithm to automatically analyze touch response videos using 2‐day‐old zebrafish. This program breaks down video files into their constituent frames which are then passed through a custom‐trained YOLOv8n algorithm to detect the presence of a single zebrafish (mean average precision, mAP50–95 = 0.74). LaZeTrack then refines the tracking data output by the model and tabulates it into an Excel spreadsheet. It then computes and extracts four relevant swim metrics: swim duration (s), swim distance (mm), mean swim velocity (mm/s), and maximum swim velocity (mm/s). LaZeTrack rapidly accelerates the analysis process, while also eliminating errors associated with manual tracking. Furthermore, it allows for high‐throughput analysis of zebrafish touch response videos and can detect subtle differences in motor metrics arising from temperature differences, demonstrating the utility of this tracking algorithm. LaZeTrack is available for download on GitHub (Armstrong‐Lab‐70/LaZeTrack).

## 1 INTRODUCTION

Zebrafish are a widely used vertebrate model organism due to their rapid development, semitransparent embryos, and accessible nervous and motor systems for investigating behavior [1]. Furthermore, the zebrafish genome shares a high degree of genetic conservation with the human genome and is amenable to editing, including by use of the CRISPR‐Cas9 system to generate both knockout and knock‐in models [2, 3, 4], making them an excellent model for biological studies. Moreover, their suitability for large‐scale genetic and small molecule screens allows for the identification and functional analysis of genes and cellular pathways involved with disease. Among vertebrate models, zebrafish have emerged as a particularly valuable organism for investigating development and function of the motor system [5, 6, 7, 8], where motor touch responses can be used to evaluate the therapeutic potential of molecules [9, 10].

The zebrafish embryo develops the ability to respond to tactile stimuli at 21 h post‐fertilization [5], where its emerging motor system changes from spontaneous coiling behavior [5] to the propulsive behavior required for hatching at 2 days of age [11]. The touch‐evoked escape response is frequently studied in 2‐day‐old zebrafish, where a light touch to the tail or head elicits burst swim behavior that lasts a few seconds [11]. The motor touch response assay is conducted by placing a single zebrafish into the center of a large Petri dish filled with system water, where burst swimming behavior is evoked by using a pair of forceps. Touch‐evoked responses are recorded from above using a video camera, using both high resolution and frame rates, enabling the data to be of sufficient quality for post hoc manual analysis [12]. Video capture and analysis software such as StreamPix5 can be used to analyze motor touch‐response assays in order to accelerate the analysis process [12]. However, high quality cameras and analysis software designed for laboratory use can be expensive and inaccessible to many researchers. Free alternative software, such as Softonic for video capture and ImageJ for analysis, provide an accessible yet imperfect solution [12]. Manual tracking using ImageJ requires frame‐by‐frame analysis and data post‐processing (i.e., extraction of relevant swim metrics from tracking data) to be performed by hand, where it is time‐consuming and prone to errors.

In this study, we developed a program named LaZeTrack with the objective of creating a tool for rapidly and reproducibly analyzing 2‐day‐old zebrafish motor touch response videos. Although numerous deep‐learning zebrafish tracking programs exist [13, 14, 15], none are available for the tracking of touch‐evoked motor responses in 2‐day‐old fish as described herein. The presence of moving distractor objects, such as the investigator’s hand and moving forceps, complicates the automation of zebrafish tracking in our specific context. LaZeTrack is intended to overhaul outdated and lengthy manual tracking approaches without the need for purchasing expensive software. This program was developed using the Python programming language on Google’s Colaboratory platform, making the code and the LaZeTrack program freely accessible to investigators. This program is centered on Ultralytics’ YOLOv8n object detection model, which is an open‐source computer vision (CV) algorithm available as a Python package [16]. Recent advances in object detection algorithms provide a rapid, accurate, and reliable analysis tool for tracking in videos [17]. In our application, the YOLOv8n algorithm is given individual video frames of 2‐day‐old motor touch response videos in order to predict the location of a single zebrafish in a sequence of video frames. Then, it refines the tracking data using a custom CV algorithm before calculating swim metrics. LaZeTrack generates an Excel spreadsheet containing four swim metrics: swim duration (s), swim distance (mm), mean swim velocity (mm/s), and maximum swim velocity (mm/s). LaZeTrack is capable of rapidly analyzing multiple videos, enabling researchers to carry out accurate high‐throughput data analysis of zebrafish motor patterns.

## 2 RESULTS

### 2.1 Workflow for motor touch response tracking and analysis

The automated tracking program (Figure 1) was designed to run on Google’s Colaboratory platform and requires that users have a free Google Drive account in order to upload videos. The program analyses grayscale motor touch response videos of individual 2‐day‐post‐fertilization zebrafish filmed at a frame rate of 30 Hz (or greater) and with a resolution of 480 × 640 pixels. The program begins by prompting users for a frame rate and an *x*–*y* calibration (*x*–*y* cal) value to relate pixel space to real space, and functions under the assumption that these two values (frame rate and *x*–*y* cal) remain constant throughout all videos in the video folder specified by the program. The program checks for erroneous user input before commencing video analysis using a custom‐trained version of Ultralytics’ YOLOv8n object detection algorithm. Then, LaZeTrack analyzes each video, generating an output comma‐separated values (.csv) file containing tracking predictions for each video. As the algorithm can make erroneous predictions, LaZeTrack also attempts to rectify any mistakes that may have been introduced during the prediction process. To further accelerate analysis, the program also creates an Excel (.xlsx) file. This file contains two sheets: one containing the tracking data from each video in the folder and another that is a summary sheet presenting four swim metrics for each video. Finally, the program also provides tracking data in individual .csv files compatible with ImageJ’s manual tracking plugin for manual review.

**FIGURE 1:**
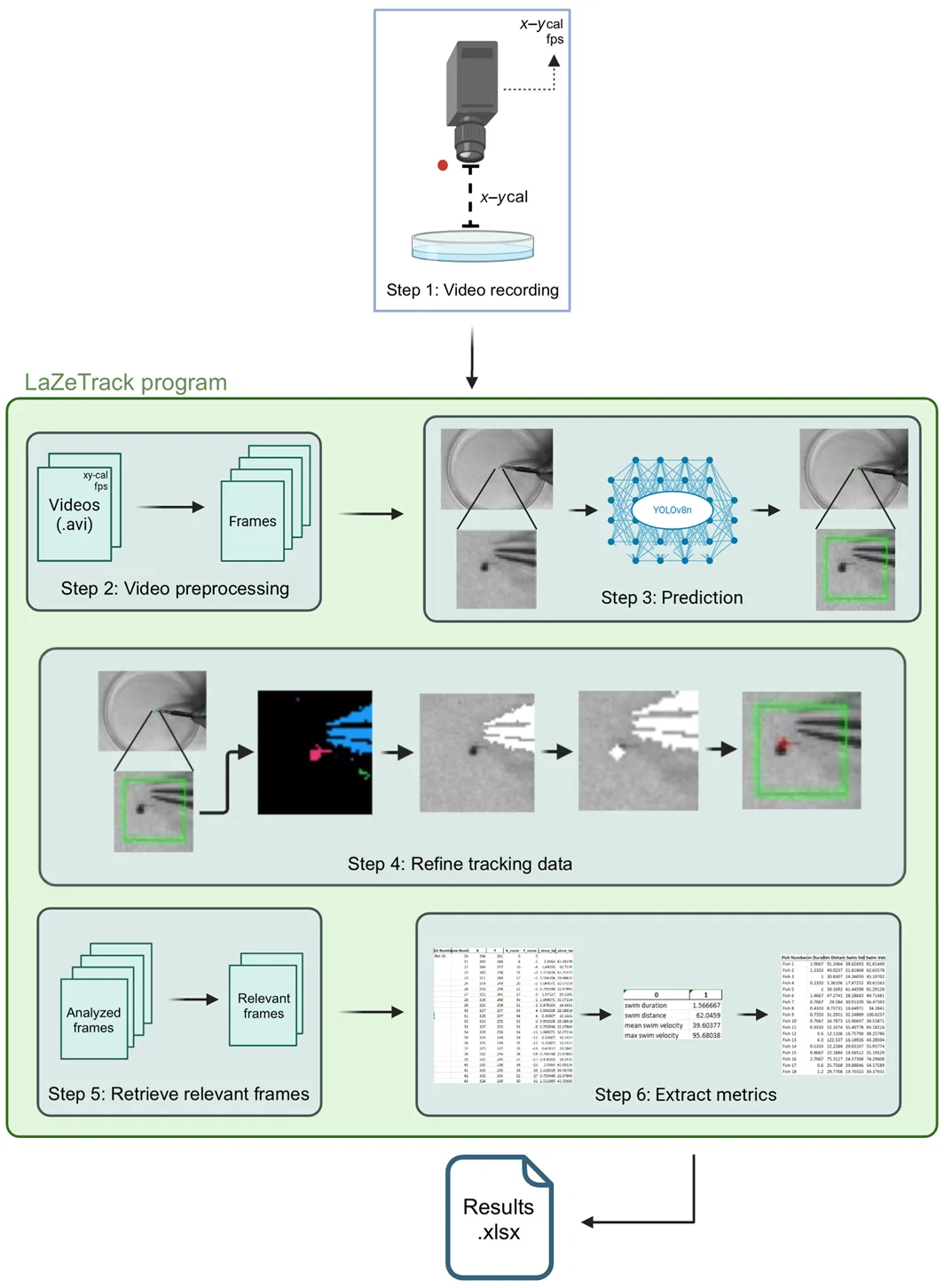
LaZeTrack application workflow. (A) Users are prompted to input a folder which contains videos of touch‐evoked motor responses of individual zebrafish. Users are required to provide an *x*–*y* calibration (*x*–*y* cal) value, obtained by dividing the width of the camera frame in meters by the width of the camera frame in pixels. This calibration value allows the tool to relate pixel distances to real‐life distances and must be adjusted every time the camera’s height is adjusted. Users are also required to provide the frame rate at which the videos were recorded. The program assumes that both the *x*–*y* cal and the frame rate values remain the same throughout a batch of input videos. (B) LaZeTrack splits recorded video sequences into individual frames for the YOLOv8n model. (C) Predictions are obtained from the model. The raw tracking data is output in the form of a bounding box, which identifies an area within which the model predicts the embryo to be present. (D) LaZeTrack applies computer vision techniques (through the OpenCV Python library) to refine the tracking data. When necessary, LaZeTrack identifies and removes distractor objects. First, the individual frame is segmented into a series of connected components. Second, the largest connected component is identified, then masked and removed from the image. This operation is performed to remove the forceps from the image. Third, the program takes the average location of the darkest pixel across all frames of a video, then masks and removes that point. This operation is performed to remove the dark, central dot marked on the Petri dish. Fourth, and finally, the location of the zebrafish head is identified as the darkest point in the bounding box. This process is repeated individually for each frame of video. (E) The refined tracking data is processed to detect the start and end of the burst swimming behavior. (F) After the tracking data has been refined and narrowed down to relevant frames, it is processed into four metrics: swim distance, swim duration, mean swim velocity and maximum swim velocity into an Excel file containing tracking data as well as swim metrics for each video.

### 2.2 Data collection and extraction

Zebrafish touch response tracking data (*n* = 1084 videos) collected from previous projects were repurposed for the creation of a training dataset. These data included .xlsx files containing tracking data, as well as corresponding videos from which the tracking data had been manually obtained in previous research. Custom Python scripts were written to extract data from .xlsx files into a YOLOv8‐compatible annotation format (Figure 2A). Another custom Python script was written for the purpose of extracting individual frames of video and matching them to the corresponding annotations (Figure 2B).

**FIGURE 2:**
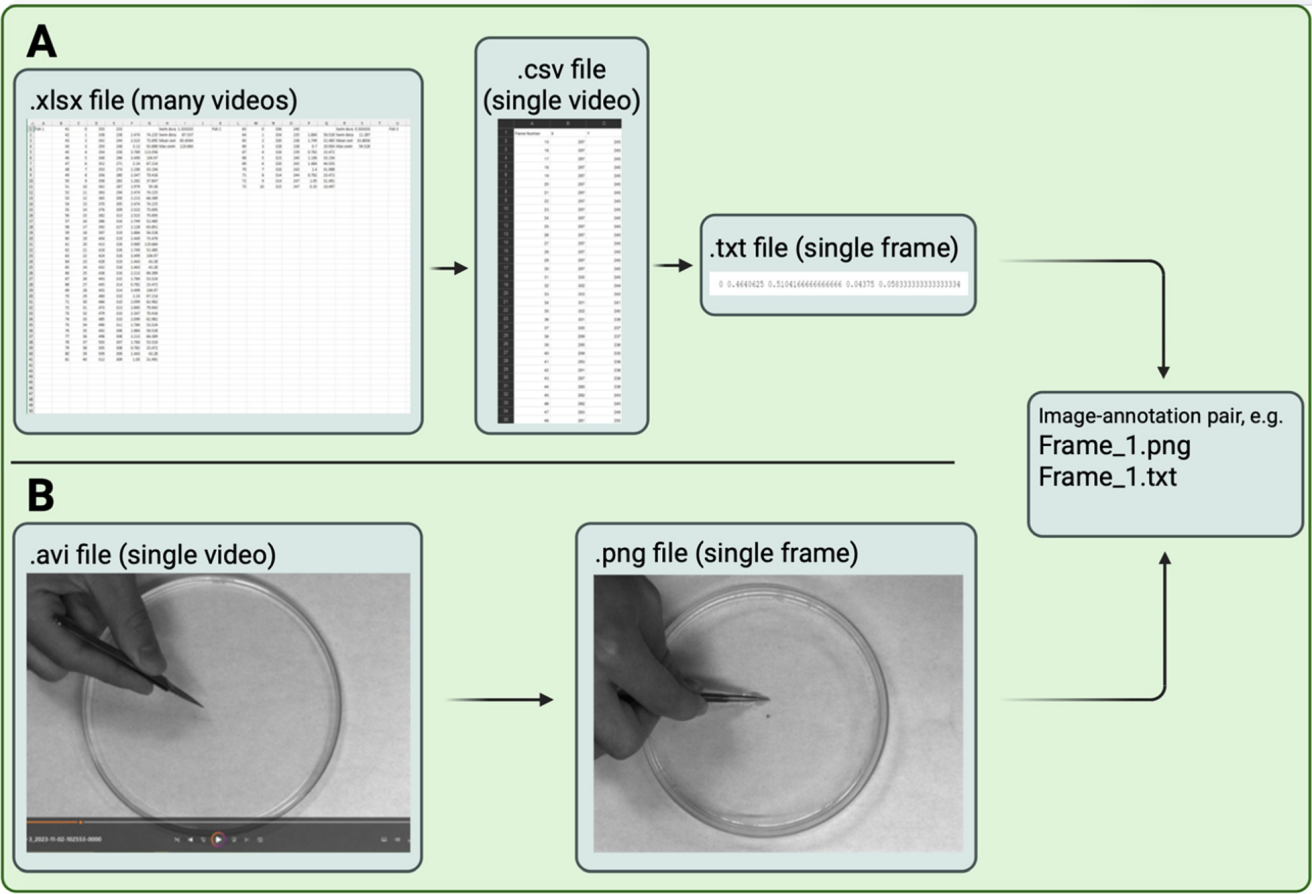
Data processing pipeline for dataset curation. (A) The process begins with matching Excel files to corresponding videos. After manual renaming of files, a custom Python script extracts image‐annotation pairs from the manual tracking data found in the Excel files and their corresponding touch response videos. Each Excel file is broken down into individual .csv files, each representing a single video. Then, the .csv files are broken down into individual text files in the YOLOv8 PyTorch .txt format. (B) Following this, the video files (.avi file format) are broken down into individual images, each named correspondingly to their matching annotation file allowing for the training using YOLOv8n.

### 2.3 Training YOLOv8n

The final dataset used for training comprised 65,416 image‐annotation pairs with annotations provided in the YOLOv8 PyTorch‐compatible .txt format. A train–test–val split (80–10–10) was applied to separate the dataset into training, testing, and validation sets. Only the training and validation sets were provided to the model during training. The pretrained YOLOv8n model was selected based on shorter training time and the relative simplicity of the detection task. The model was trained using an NVIDIA GeForce RTX 2070 Super (8192 Mb) GPU.

The pretrained YOLOv8n model was trained for 400 epochs with a batch size of 8 and 4 data‐loading workers, using early stopping with a patience of 30 epochs. Input images were resized to 640 pixels × 640 pixels. Optimization was performed using the default YOLOv8 optimization settings (stochastic gradient descent with momentum), with an initial learning rate of 0.01, momentum of 0.937, and weight decay of 5 × 10^−4^. Learning rate warmup was applied for the first 3 epochs. Mixed‐precision training was enabled, and deterministic behavior was enforced.

Default YOLOv8 data‐augmentation strategies were applied, including mosaic augmentation, random scaling, translation, horizontal flipping, color jitter, and RandAugment. Unless otherwise specified, all remaining hyperparameters were left at their default values.

### 2.4 YOLOv8n algorithm

The model used to develop our zebrafish tracking program was Ultralytics’ YOLOv8n [16]. Although the performance of the nano model is limited due to its relatively low number of parameters (2 million), it is still well‐suited for simple object detection tasks that must run in computationally constrained environments. Therefore, this model provides an accessible object detection algorithm that performs well without requiring expensive computer hardware. Briefly, the algorithm can be broken down into three modules: a backbone, a neck, and a head [16, 17, 18]. The backbone module consists of a convolutional neural network which extracts multi‐scale features from input images [17]. The neck module is a multi‐scale feature integrator, which refines the features extracted by the backbone and fuses them in a process which is required for detecting objects of varying shapes and sizes [17]. The head module generates the final predictions, including class labels, confidence scores, and bounding box coordinates for each detected object [17].

### 2.5 YOLOv8n performance with varying training dataset sizes

Although there are many performance measures for object detection models, mean average precision (mAP) is frequently used as a metric to assess overall performance [19, 20, 21]. Ultralytics’ mAP50–95 metric encapsulates both precision and recall, at an intersection‐over‐union threshold of 50%–95%, across all classes [16].

As the data were collected over the course of the project, the model was trained several times using datasets of increasing size. The performance of the model using the first dataset of 18,189 images achieved a mAP50–95 value of 0.74. When the size of the dataset was increased to 20,014 images, the model attained a mAP50–95 value of 0.79. However, on the final dataset of 65,416 images, we observed a decrease in performance to an mAP50–95 value of 0.74. These mAP50–95 values were obtained using Ultralytics’ built‐in “.val()” method, by specifying the custom‐trained model version as well as the dedicated test dataset, which was novel to the model. By using the test dataset, which constitutes 10% of the entire dataset, we ensured that the obtained performance metrics were representative of the model’s performance on unseen data, rather than data used during training such as the training and validation datasets.

### 2.6 Prediction generation and refinement

After receiving the appropriate user input, LaZeTrack reads through each video frame‐by‐frame, feeding each frame to the custom‐trained YOLOv8n algorithm. The algorithm outputs predictions (bounding boxes) for each frame, which are then saved in a .csv file that represents all the predictions for each frame of a given video (see Video S1). Once all videos have been analyzed using YOLOv8n, the program refines the predictions. As YOLOv8n outputs bounding boxes that may be centered on random points along the zebrafish body, LaZeTrack was designed to use a simple CV algorithm to refine the model’s predictions. This simple CV algorithm works as follows: (1) detect and mask a dark central dot, if present within the bounding box region; (2) detect and mask forceps used to evoke motor touch responses, if present in the bounding box region; and (3) find the local minimum pixel brightness value in the bounding box region (which is assumed to be the zebrafish head). This simple algorithm works to refine the bounding box predicted by YOLOv8n to a single Cartesian point within the image, which represents the location of the fish head. Briefly, the logic of the algorithm is based on heuristic assumptions, which rely on the highly standardized nature of the touch‐response videos. It assumes that the zebrafish head is the darkest point in each bounding box, which is true except in two cases. The first case involves the presence of a dark, central dot on the Petri dish that researchers use to initially position zebrafish in the center of the dish. To determine the location of the centering dot, the algorithm performs a search for the darkest point in every video frame and applies a mask to the average location of the darkest pixel. Importantly, the program functions under the assumption that there will always be a central dot, as is the case for all the videos the YOLOv8n algorithm was trained on. The second case involves the presence of forceps, used to evoke motor behavior by lightly touching the tail. The algorithm applies a function from the OpenCV Python library to detect the largest connected component currently in the frame and applies a mask to this object. In cases where both the dot and forceps are present, the algorithm applies both masks. In cases where the position of the head cannot be reliably determined, the default value is the center of the bounding box. Later steps in the program review the predictions in order to remove erroneous predictions.

### 2.7 Prediction correction

Although the custom‐trained YOLOv8n model performs well on zebrafish detection tasks, it can make prediction errors. For example, the model may detect two fish when only one is present in a frame (note: LaZeTrack can only be used to track single‐zebrafish touch responses), or it may erroneously predict the single zebrafish position, or fail to generate any predictions for a given frame. Although these errors are uncommon, LaZeTrack addresses them by searching through each .csv file generated by the prediction step. This process detects errors through three main approaches. (1) If the program finds failed predictions in the first few or the last few frames, it removes them from the .csv file, as these frames usually do not contain zebrafish movement. (2) It verifies that each velocity value is realistic; if a zebrafish’s velocity has exceeded 300 mm/s over the last frame, the prediction is deleted and replaced with an empty value. (3) If empty values are found, after the previous two steps have been completed, they are assumed to be failed detections and are linearly interpolated. This is achieved by linearly interpolating the zebrafish’s position using the previous frame and the following frame, as this preserves a sufficient amount of information about the swim pattern. Because the error correction process functions under a number of assumptions, it is possible that LaZeTrack can fail to properly address errors. LaZeTrack thus also generates additional .csv files that contain the predicted locations of the zebrafish in each frame for each video. These .csv files have been formatted to be compatible with ImageJ’s “manual tracking” plugin, allowing users to manually review LaZeTrack’s output for errors.

### 2.8 Extraction of relevant frames

Given that the YOLOv8n algorithm functions solely to locate objects within an image, the machine learning (ML) algorithm itself cannot determine when a fish’s movement pattern begins or ends. Therefore, LaZeTrack takes a simple thresholding approach to detect the start and end of swim patterns. To detect the start of a burst swim pattern, LaZeTrack applies a One Euro filter to the predicted positional data and sets a threshold for movement initiation at 30 mm/s. The One Euro filter is designed for smoothing time series data. It functions as an adaptive low‐pass filter, adjusting its smoothing strength based on the rate of change in the signal (e.g., the position of the zebrafish head) [22]. When the signal changes slowly, the filter applies stronger smoothing to reduce noise, whereas when the signal changes rapidly, it decreases smoothing to preserve responsiveness. To achieve this dynamic filtering, the One Euro filter applies a standard exponential moving average with a dynamic cutoff frequency that varies according to the signal’s derivative [22]. To detect the end of a swim pattern, we apply the One Euro filter to the positional data and calculate a velocity value. When the zebrafish’s velocity dips below the threshold value of 15 mm/s, the swim pattern is considered terminated. Although the thresholding approach described here functions robustly, it can malfunction and result in erroneous start and end times. For example, we conducted an analysis on data obtained manually and data obtained by LaZeTrack and observed a slight trend toward prematurely detecting the beginning of swim patterns (Figure 3A). In contrast, the program performs robustly at detecting the end of swim patterns (Figure 3B).

**FIGURE 3:**
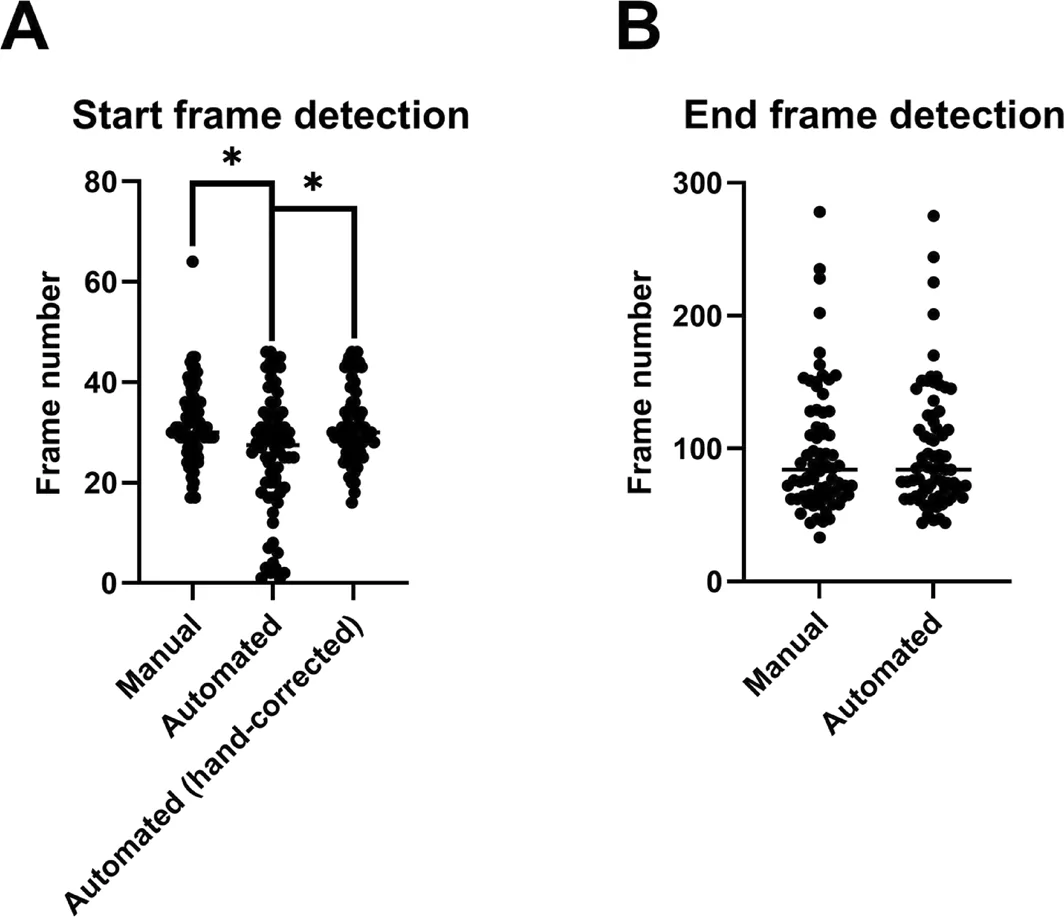
LaZeTrack adequately detects the start and end of 2‐day‐old zebrafish swim patterns. (A) LaZeTrack program tends to prematurely detect the start of swim patterns. The manual group shows the frames at which the start of swim patterns was detected, with each data point representing one video. Automated detection is significantly different from manual detection of swim pattern starts (*p* = 0.016), but with manual correction the data is not significantly different. (B) LaZeTrack program accurately detects the end of the swim patterns, as evidenced by the lack of a significant difference in the stop frames detected by the program. Sample sizes are as follows: All groups, *n* = 72. Significance was assessed using Kruskal–Wallis test for start frame groups, and Mann–Whitney *U*‐test was used for end frame groups. Significant differences are denoted as: *^∗^p* < 0.05.

### 2.9 Analysis of tracking data

Another challenge faced by researchers utilizing the zebrafish motor touch response assay is the analysis of large amounts of tracking data (e.g., drug or genetic screens). Conventionally, this required the manual import of output data into a spreadsheet for each video recorded. Then, researchers had to manually add formulas to each video’s data in order to generate four swim metrics—swim duration (s), swim distance (mm), mean swim velocity (mm/s), and maximum swim velocity (mm/s)—thereby creating complete datasets. Although less challenging than tracking individual fish touch responses, this post‐tracking analysis requires time. Therefore, we included built‐in analysis of tracking data as a feature of LaZeTrack. The program uses the user‐specified value for *x*–*y* cal to relate pixel space to real space. The *x*–*y* cal value can be obtained by measuring an object in frame (e.g., the diameter of the Petri dish) using the measuring tool in ImageJ and dividing the object’s length in millimeters by its length in pixels. Thus, the user obtains an *x*–*y* cal value in units of millimeter‐per‐pixel. The LaZeTrack program functions under the assumption that all the videos in a batch are recorded with the same *x*–*y* cal value. In instances in which the *x*–*y* cal changes (e.g., distance of the camera lens to the Petri dish) users will have to change the *x*–*y* cal value by analyzing a separate batch of videos separately. The program calculates swim duration by dividing the total number of frames by the frame rate. Swim distance is calculated by taking the sum of all cells in the “distance_since_last_frame” column, which is generated by taking the Cartesian distance between the current and previous points and multiplying the result by the *x*–*y* cal value. Mean swim velocity is calculated by dividing the “distance_since_last_frame” value by the frame rate for each cell and then averaging all values in the swim pattern to obtain the mean swim velocity. Maximum swim velocity is calculated by finding the maximum value in the “velocity” column. In the final step, LaZeTrack takes each of the four swim metrics for each fish in the batch and generates a summary page. This page shows each fish’s four swim metrics, as well as the mean for each metric across all zebrafish in the batch and outputs an .xlsx file.

### 2.10 Detection of zebrafish phenotypes

In order to determine the ability of the LaZeTrack program to detect subtle motor phenotypes, we conducted a simple assay in which zebrafish were subjected to various temperatures and allowed us to investigate the effect of temperature on motor function. We selected three temperatures to investigate. Zebrafish were raised at the normal rearing temperature of 28.5 °C, but 1 h prior to the motor touch assay zebrafish were switched to system water at a temperature of either 23.5 °C, 28.5 °C, or 33.5 °C. We hypothesized that elevated water temperature would result in zebrafish displaying increased swim duration (s), swim distance (mm), mean swim velocity (mm/s), and maximum swim velocity (mm/s). Conversely, we hypothesized that decreased water temperature would result in lower values of our four motor function metrics. Although swim duration was not significantly altered in the three temperature groups (Figure 4A), we observed that swim distance was significantly increased in both the 28.5 °C and 33.5 °C groups when compared with the 23.5 °C group (Figure 4B). In addition, LaZeTrack detected an increase in mean swim velocity in zebrafish incubated at 33.5 °C when compared to zebrafish incubated at 23.5 °C (Figure 4C). Furthermore, LaZeTrack also detected an increase in maximum swim velocity in the 28.5 °C and 33.5 °C groups when compared to the 23.5 °C group (Figure 4D). Taken together, these results suggest that LaZeTrack is capable of robustly detecting subtle changes in zebrafish motor touch responses.

**FIGURE 4:**
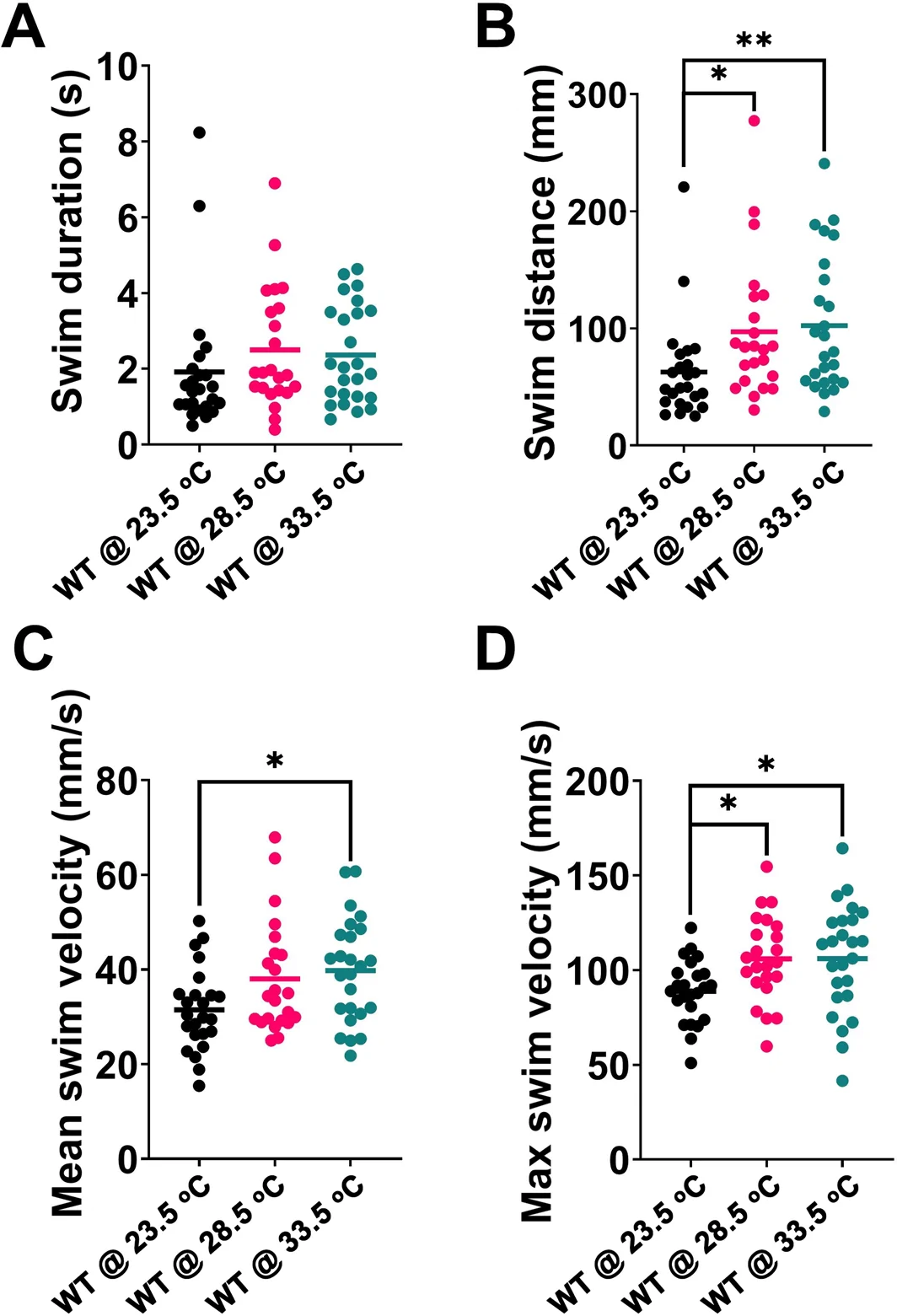
LaZeTrack captures temperature‐dependent alterations in motor function of 2‐day‐old zebrafish touch response videos. (A) No statistically significant increases in swim duration were observed using LaZeTrack. (B) Swim distance derived from LaZeTrack datasets captured that zebrafish incubated at both the 28.5 °C (*p* = 0.018) and 33.5 °C (*p* = 0.01) were significantly increased when compared to fish held at 23.5 °C. (C) LaZeTrack identified a statistically significant increase in mean swim velocity in the 33.5 °C group when compared to zebrafish held at 23.5 °C (*p* = 0.027). (D) LaZeTrack identified a statistically significant increase in maximum swim velocity in zebrafish held at 28.5 °C (*p* = 0.035) and 33.5 °C (*p* = 0.03) when compared to fish held at 23.5 °C. Samples sizes are as follows: 23.5 °C group, *n* = 24; 28.5 °C group, *n* = 23; 33.5 °C group, *n* = 25. Significance was assessed using Kruskal–Wallis test with post hoc Dunn's test for swim duration, swim distance and mean swim velocity, and one‐way ANOVA with post hoc Tukey’s test was used for maximum swim velocity. Significant differences are denoted as: *^∗^p* < 0.05; ^∗^^∗^ *p* < 0.01. ANOVA, analysis of variance; WT, wild type.

### 2.11 Performance compared to manual tracking

To compare our findings obtained using LaZeTrack, we analyzed the temperature ramp touch response data by hand using the ImageJ’s manual tracking plugin. Although manual analysis is subject to experimenter bias and error, it remains the standard of analysis for such videos. We hypothesized that LaZeTrack’s results would capture similar findings in our datasets. Similar to LaZeTrack, manual tracking did not reveal any significant differences in swim duration across the three temperature groups (Figure 5A). Manual tracking also showed statistically significant increases in swim distance in the 28.5 °C and 33.5 °C groups when compared to the 23.5 °C group (Figure 5B). Notably, manual tracking did not reveal any significant differences in mean swim velocity (Figure 5C). We observed a statistically significant increase in maximum swim velocity in both the 28.5 °C and 33.5 °C groups when compared to the 23.5 °C group (Figure 5D). Taken together, these results demonstrate that all significant differences observed using the ImageJ manual tracking plugin were also observed using LaZeTrack. Furthermore, to ascertain the differences between manual analysis and ML‐based tracking, we compared manual tracking data to data obtained using LaZeTrack for the 28.5 °C group (Figure 6). We observed no statistically significant differences between the two datasets, indicating that LaZeTrack adequately captures zebrafish motor touch response metrics. Although both swim duration (Figure 6A) and swim distance (Figure 6B) had similar values between manual analysis and ML‐based analysis, we observed a slight trend toward decreased mean swim velocity values in manual analysis when compared with the data captured by LaZeTrack (Figure 6C). Conversely, we also observed a slight trend toward increased maximum swim velocities in manual tracking when compared with the data captured by LaZeTrack (Figure 6D). In summary, these findings indicate that the data captured by LaZeTrack does not deviate significantly from manual tracking data, indicating its robustness as a replacement for labor‐intensive ImageJ’s manual tracking.

**FIGURE 5:**
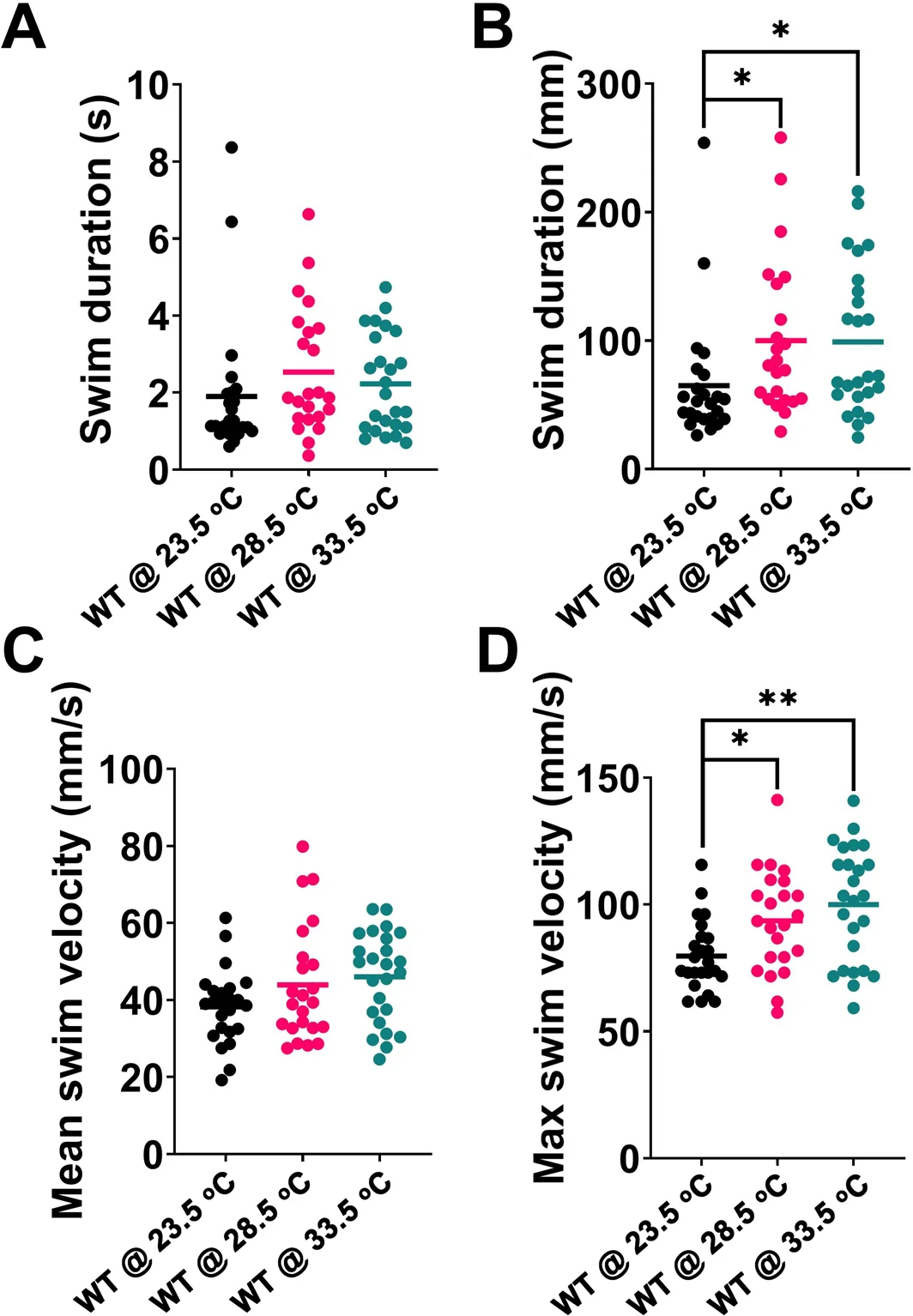
Manual analysis using the ImageJ manual tracking plugin captures temperature‐dependent alteration of zebrafish motor function from the same data as in Figure 4. (A) No significant differences in swim duration were observed across the three temperature groups. (B) Zebrafish incubated at both the 28.5 °C (*p* = 0.019) and 33.5 °C (*p* = 0.023) displayed significantly increased swim distance when compared to fish held at 23.5 °C. (C) Manual analysis did not reveal any statistically significant differences in mean swim velocity. (D) Statistically significant increases in maximum swim velocity in zebrafish held at 28.5 °C (*p* = 0.042) and 33.5 °C (*p* = 0.001) were observed when compared to zebrafish held at 23.5 °C. Sample sizes are as follows: 23.5 °C group, *n* = 24; 28.5 °C group, *n* = 23; 33.5 °C group, *n* = 25. Significance was assessed using Kruskal–Wallis test with post hoc Dunn’s test for swim duration, swim distance and mean swim velocity, and one‐way ANOVA with post hoc Tukey’s test was used for maximum swim velocity. Significant differences are denoted as: *^∗^p* < 0.05; ^∗^^∗^ *p* < 0.01. ANOVA, analysis of variance; WT, wild type.

**FIGURE 6:**
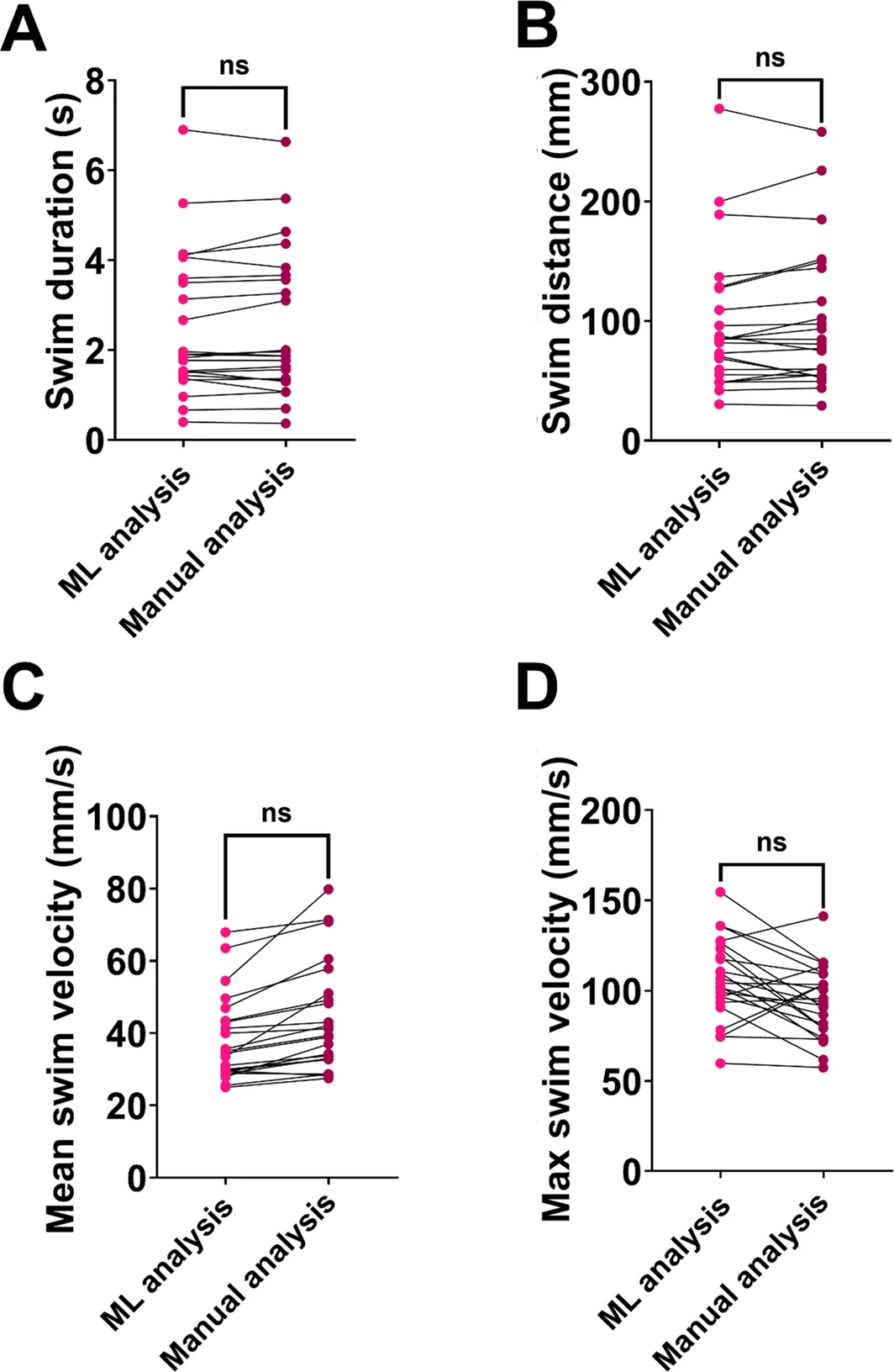
ML analysis does not significantly alter data compared to manual analysis. The data presented in this figure is from the 28.5 °C group, as analyzed manually and by the LaZeTrack program. (A) ML analysis does not significantly alter the data obtained for swim duration when compared to manual analysis. (B) ML analysis does not significantly alter the data obtained for swim distance when compared to manual analysis. (C) ML analysis tends to decrease the values for mean swim velocity when compared to manual analysis but does not significantly alter the data. (D) ML analysis tends to increase the values for maximum swim velocity when compared to manual analysis but does not significantly alter the data. Samples sizes are as follows: 28.5 °C group, *n* = 23. Significance was assessed using Mann–Whitney *U*‐test for swim duration, swim distance and mean swim velocity, and Welch’s *t*‐test for maximum swim velocity. No significant differences were identified. ML, machine learning.

## 3 DISCUSSION

In this study, we introduce LaZeTrack, an accessible, fully automated pipeline for the analysis of 2‐day‐old zebrafish motor touch response videos. Using a custom‐trained YOLOv8n object detection model combined with bespoke error correction and post‐tracking analysis, the program allows for accurate high‐throughput analysis of motor touch response data without the need for expensive software or extensive manual labor. Our results demonstrate that LaZeTrack performs comparably to manual tracking zebrafish swim metrics across experimental groups, validating its use as a reliable tool for behavioral phenotyping in this narrow task.

A key strength of LaZeTrack lies in its scalability and accessibility. The use of Google’s Colaboratory platform and free file‐hosting through Google Drive ensures that researchers without access to expensive specialized hardware or proprietary software can still efficiently perform large‐scale behavioral analyses. Moreover, the use of the YOLOv8n model further reinforces this accessibility, as it allows for rapid analysis of motor touch response videos even on nonspecialized computing hardware. Although larger models may offer marginal gains in accuracy, our results suggest that YOLOv8n provides sufficient precision for the relatively simple task of single‐object tracking in standardized video conditions.

LaZeTrack serves as an example that even with limited data, it is possible to achieve high performance on a CV task using the YOLOv8n model. Although we hypothesized that performance (as measured by mAP50–95 on separate, unseen test datasets) would increase with larger amounts of training data, we observed the opposite trend; the mAP50–95 value for the first dataset of 18,189 images was 0.74. With an increase to 20,014 images for the second dataset, a mAP50–95 value of 0.79 was achieved. However, on the final dataset of 65,416 images, we observed a decrease in mAP50–95 to a value of 0.74. There are multiple interpretations of this decrease in performance. For example, it is possible that the largest dataset, which contained a wider range of data, was more variable than smaller datasets. Thus, it may have captured a more realistic picture of the variability inherent to touch response videos collected by different researchers. For example, various distractor objects such as a floating particulate, hands, and forceps may occasionally occlude fish in videos, zebrafish pigmentation may vary based on their genotype or treatment, and experimenters may have slightly different techniques for evoking the motor responses, resulting in different situations for the model to interpret. By providing a larger dataset, we provide a more realistic snapshot of what future videos may look like, thus providing the model with a more robust training scope despite the slight reduction in overall performance. Conversely, it could be argued that a smaller amount of training data was sufficient to achieve usable performance for this task, and that adding more data was not beneficial to performance. Nevertheless, our findings suggest that the pre‐training provided with YOLOv8n is sufficient to offset the effects of limited datasets, allowing this model to be used even in circumstances when researchers would like to implement a ML solution without an abundance of training data.

To demonstrate the utility of LaZeTrack, we examined the effect of temperature on motor touch responses and compared these with manual tracking results obtained from the same datasets. We observed that LaZeTrack reliably detected subtle differences in motor function metrics among touch responses, as evidenced by its ability to detect similar trends to those detected by the current standard of tracking zebrafish motor patterns (ImageJ’s manual tracking). Although the two methods for tracking metrics of motor function were similar, we observed a statistically significant increase in mean swim velocity between the 23.5 °C and the 33.5 °C groups in LaZeTrack’s results, whereas manual analysis failed to reveal any such difference (Figures 4C and 5C). Given these results, we theorized that the ability of LaZeTrack to robustly track a single location on the zebrafish (i.e., the zebrafish head) provides more accurate, less noisy tracking data than error‐prone manual analysis, thus reducing Type II errors.

During the development of LaZeTrack, two issues routinely encumbered the program, which were detecting zebrafish swim pattern initiation and termination (henceforth referred to as zebrafish starts and stops, respectively). To overcome these issues, we integrated a One Euro filter and a velocity thresholding strategy, thereby allowing LaZeTrack to successfully identify the start and end of zebrafish swim patterns (Figure 3). The One Euro filter is commonly used in applications requiring real‐time tracking, such as motion capture, human‐computer interaction, and robotics [22]. Although our application does not involve real‐time tracking, the same principles apply when capturing swim patterns. The thresholding approach described herein robustly detects zebrafish starts and stops, but is subject to certain limitations. On one hand, the detection of zebrafish starts can be mired by the presence of distractor objects lowering tracking fidelity and thus disrupting our thresholding approach. On the other hand, detection of zebrafish stops is complicated by their inherent variability, owing to the gradual deceleration and the noisier nature of tracking data at low speeds. Although these limitations mirror the ambiguity often encountered in manual analysis, improving the zebrafish start and end detection algorithm represents a direction for future iterations of algorithmic approaches to tracking zebrafish swim patterns. Incorporating ML‐based temporal classifiers or recurrent models could help increase the reliability of start and end detections compared with the thresholding approach currently taken by LaZeTrack.

Importantly, LaZeTrack’s ability to distinguish temperature‐dependent differences in swim metrics demonstrates its practical utility in behavioral assays. The detection of statistically significant changes in swim distance, mean velocity, and maximum velocity in zebrafish exposed to elevated temperatures is consistent with known effects of temperature on zebrafish physiology [23, 24]. These findings were further corroborated by manual tracking, which revealed similar patterns to the tracking data provided by LaZeTrack. Our results support the use of LaZeTrack as a robust, user‐friendly, and cost‐effective alternative to the ImageJ’s manual tracking. Given that the start and end detection capabilities of the program remain the main limitation of LaZeTrack, albeit a minor limitation, we suggest that users of LaZeTrack manually review the data output by the program. Nonetheless, the benefits of LaZeTrack’s automation, consistency, and accessibility make it a valuable tool for the zebrafish research community. Future developments could include integration with a graphical user interface, improved zebrafish start and end detection, or deep learning‐based tracking. Although the current pipeline focuses on single‐animal tracking using motion‐constrained association, future iterations of LaZeTrack could integrate modern tracking‐by‐detection frameworks such as ByteTrack or DeepSORT. These methods may be particularly advantageous in multi‐animal assays or more complex arenas where identity maintenance and occlusion handling become critical. In addition, a subsequent version of LaZeTrack could be adapted for use in older larval zebrafish when they have re‐organized their spinal cord motor Circuitry to a beat‐and‐glide motor pattern. In summary, LaZeTrack reduces analysis time for the 2‐day‐old motor touch‐response assay from hours to seconds, and thus it may accelerate the pace of discovery in future studies using zebrafish.

## 4 MATERIALS AND METHODS

### 4.1 Zebrafish husbandry

Adult zebrafish (*Danio rerio*) were maintained and bred according to standard protocols under a 14/10 light/dark cycle at 28.5 °C [25]. All experiments were performed in compliance with the guidelines of the Canadian Council for Animal Care at the Montreal Neurological Institute at McGill University (Montreal, Quebec, Canada) and approved by the Animal Care Committee (AUP number: MNI‐7890).

### 4.2 Zebrafish touch responses

The touch‐evoked motor response assay was performed as previously described [26]. Zebrafish were placed in the center of a circular dish (150 mm in diameter) containing system water at approximately 1 cm in depth. Burst swimming behavior was evoked by a brief touch to the tail, and the swim pattern was recorded from above at 30 Hz using a digital camera (Grasshopper 2 camera; Point Gray Research). Swim duration, distance, mean, and maximum velocities were quantified using the manual tracking plugin for ImageJ (version 1.54j). Additional details pertaining to the methods for recording and manually tracking touch‐evoked motor responses can be found in the Supplementary Methods. For the purposes of quantifying YOLOv8n model tracking performance, these metrics were also analyzed based on tracking data obtained automatically from the model’s predictions.

### 4.3 Statistical analysis

All statistical comparisons were performed using Prism 10 (GraphPad Software Inc.). Data are presented as mean ± standard error. A Shapiro–Wilk test was used to assess normality. When data were normally distributed, a one‐way analysis of variance followed by Tukey’s multiple comparisons test, or Welch’s *t*‐test, was performed to identify where differences lay. When data were not normally distributed, a Kruskal–Wallis test followed by Dunn’s multiple comparisons test, or Mann–Whitney *U*‐test, was performed to identify where differences lay. Significance was assessed at *p* < 0.05.

## AUTHOR CONTRIBUTIONS

**Adrien Lacroix**: Conceptualization; data curation; formal analysis; investigation; methodology; software; validation; visualization; writing—original draft; and writing—review and editing. **Gary Alan Barclay Armstrong**: Funding acquisition; project administration; resources; supervision; writing—original draft; and writing—review and editing.

## CONFLICT OF INTEREST STATEMENT

The authors declare no conflicts of interest.

## ETHICS STATEMENT

All experiments were performed in compliance with the guidelines of the Canadian Council for Animal Care at the Montreal Neurological Institute at McGill University (Montreal, Quebec, Canada) and approved by the Animal Care Committee (AUP number: MNI‐7890).

## Supporting information

Supporting Information S1

Supporting Information S2

Video S1

## Data Availability

The code and data underlying this research are available on GitHub (Armstrong‐Lab‐70/LaZeTrack) as well as on Figshare DOI:10.6084/m9.figshare.29631308. Please note that, due to file size restrictions, our training datasets for YOLOv8n cannot be published online but will be shared upon reasonable request.
