# Supplementary material for: LaZeTrack: An open‐source object detection algorithm to detect and track 2‐day‐old zebrafish motor touch responses
Source: Quant Biol. 2026 Jun 1;14(3):e70043. doi: 10.1002/qub2.70043 (PMC13642170; doi:10.1002/qub2.70043)
Supplement: Supplementary file 1 [file QUB2-14-e70043-s001.docx]

**Supplemental Video 1**

**Supplemental Video 1.** An example video of a zebrafish exhibiting the touch-evoked motor response, overlayed with YOLOv8n’s tracking predictions.
