# Supplementary material for: LaZeTrack: An open‐source object detection algorithm to detect and track 2‐day‐old zebrafish motor touch responses
Source: Quant Biol. 2026 Jun 1;14(3):e70043. doi: 10.1002/qub2.70043 (PMC13642170; doi:10.1002/qub2.70043)
Supplement: Supplementary file 2 [file QUB2-14-e70043-s002.docx]

**2-day old Zebrafish Motor Touch Response Protocol**

This is the protocol outlining best practices for the zebrafish motor touch response. Current literature suggests that the best time window during which to record these responses is 52 h to 54 h post-fertilization (i.e., 12PM-2PM two days after you collect your eggs).

**Two Days Before:**

Collect the eggs from your cross, ensuring to **store them in bottles with system water, NOT methylene blue system water** (Methylene blue is neuroprotective and may skew results).

**Day of Experiment:**

**10:00AM:** Begin dechorionating your zebrafish embryos, making sure not to harm them (e.g., by breaking their tails, etc.).

**11:00AM:** Set up the hot plate. Do this at least 30 minutes before you want to start recording.

Materials:

- Hot plate
- (optional) Cardboard sheet, for a white background (if the hot plate isn’t white)
- 13.8 cm petri dish
- Digital thermometer (found in the black case on a shelf above the recording setup)

Methods:

1. Place the hot plate directly underneath the Grasshopper camera.
   a. (optional): place the cardboard sheet on the hot plate
2. Fill the petri dish with 0.5–1 cm of water. The water should be high enough so that the larvae can swim around, but also low enough that they aren’t swimming up and down within the water column.
3. Place the petri dish on top of the hot plate, making sure it is centered on the element.
4. Turn on the hot plate, setting it to 31.5 °C (every hot plate is different so you may have to change this).
5. Take the thermometer out of its case and plug in a probe wire (make sure that the probe is plugged in so that + ve and − ve are in the correct orientation).
6. Turn on the thermometer and take a reading by moving the probe around in the water. Do this intermittently over 30 minutes, after this the water should be around 24 °C to 25 °C.
   a. You will notice a temperature gradient establish itself across the dish. The center of the dish will be reliably 1 °C to 2 °C cooler than the edges. To address this, aim for 24 °C at the center of the dish, that way the edges don’t get too warm.

0

7. Repeat step 6 until the water has reached the correct temperature.

**11:50AM:** Set up the camera and computer for recording. This can be done at any time as it only takes a few minutes.

Methods:

1. Turn on the computer, and log in using the password specified on the monitor.
   a. If you haven’t already, create a folder on the desktop where you can store your videos, and title it as your initials.
   b. Create a sub-folder within your desktop folder where you will store the videos you record today. Name it after the type of larvae you will be recording and the date (e.g., “WT Touch Response dd-mm-yyyy”).
2. Open the “Point Grey FlyCap2 software”. It can be found on the taskbar (logo resembles a blue “C”), or on the search bar by typing “Point”.
3. Under “Camera List”, click on “Grasshopper GRAS-03K2M” so that it is highlighted in blue, and then press configure selected.
   a. In the pop-up menu on the left, click “Standard Video Modes”
   b. Under “Frame Rate” on the right, click 30.00 Hz.
   c. Close this pop-up window
4. Click on “OK” on the original Point Grey FlyCap2 screen. This will open the camera.
5. On the camera screen, verify that “Frame Rate” is reading at 30.00 fps.
6. Focus the camera (N.B. you will need to put a larva in the dish to do this):
   a. On the physical camera (not on the computer), there are two rotating segments. The bottom segment controls focus.
   b. Rotate this segment until the larva in the dish is in sharp focus.

**11:55AM:** Set up your dishes under the microscope for easy access to the larvae while you record.

Materials:

- Large petri dish
- Small petri dish
- System water

Methods:

1. Retrieve your bottles from the incubator, where they should have now spent at least an hour (to allow the zebrafish to straighten out after dechorionation).
2. Into a larger petri dish, pour the contents of the bottle. The large petri dish is where you will place your zebrafish and record one fish at a time.
3. Fill a second petri dish with system water. The small petri dish is where you will put your zebrafish after recording their motor touch response.

**12:00PM:** Begin recording touch responses.

Materials:

- Forceps
- Transfer Pipette

Methods:

1. Press the red record button on the FlyCap2 camera screen.
2. Under “save filename”, press browse and select the video folder you created earlier (should be under “Desktop/YourFolder”).
3. Under “saving options”, press the “Capture for ____ ms” option, and type 10000 ms (10s).
4. Click to reveal the “Videos” tab.
   a. Choose M-JPEG as the file format.
   b. Set the frame rate to 30 fps by clicking “use camera frame rate”.
   c. Set the compression quality to “100”.
5. Set up the zebrafish in the dish.
   a. Under the microscope, scan your petri dish for the zebrafish. Choose only the properly developed zebrafish with no morphological defects, and with full pigmentation (hypopigmented zebrafish should not be used).
   b. Using the transfer pipette, extract one healthy zebrafish and gently deposit it in the middle of the 13.8 cm petri dish.
   c. If the zebrafish begins to swim, use the transfer pipette to place it in the center of the dish again.
   d. Repeat step 5c until the fish remains still for approximately 20 seconds.
6. Record.
   a. Using the forceps, position your hand so that you are ready to execute a smooth, steady swiping motion across the center of the dish which will touch the zebrafish’s tail.
   b. Click the “Start Recording” button in FlyCap2.
   c. Execute the tail touch, removing your hand as soon as possible without occluding the fish.
   d. Click “Stop Recording” once the fish stops moving or the time runs out.
   e. Using the transfer pipette, place the zebrafish in the small petri dish (for “used” fish).
7. Repeat steps 5 and 6 until you have recorded all your videos.

**Tracking Instructions**

Next, you must manually track the position of your touch response videos that you recorded. This is done using ImageJ’s (available at: [imagej.net/ij](https://imagej.net/ij/)) manual tracking plugin (available at: [imagej.net/ij/plugins/track/track](https://imagej.net/ij/plugins/track/track.html)).

Once you have installed ImageJ and the manual tracking plugin, open ImageJ, and perform each of the following steps for each video you wish to analyze:

1. Open the video folder in your file manager (e.g., Finder on Mac, or File Explorer on windows)
2. Drag and drop the video into your open instance of ImageJ
3. The video should pop up. Now, in the menu at the top of ImageJ, click the Plugins tab, and click Manual Tracking.
4. The Manual Tracking plugin window should pop up. At the bottom, you will find the parameters.
5. Modify the parameters as follows (if not present here, leave at default value):
   - Time interval: 0.3333 sec (for 30 Hz video)
   - x/y calibration: Use the “Measure tool” under the “Analyze” tab in the menu to measure the width of the petri dish in frame. Divide the width in mm by the width in pixels to obtain your xy-cal value. It should be approximately 0.3–0.35.
   - Dot size: 1.0
6. Now you are ready to track. Press the “Add track” button to begin a new track.
7. Use the arrow keys to navigate through the first few frames of video. You want to begin your track the first frame BEFORE the fish moves. Find this frame.
8. Click the fish’s head to add a tracking point. Repeat until the zebrafish stops swimming (do not track multiple swim patterns).
   - TIP: use the “Delete last point” button to delete the last point you added if you misclick.
9. When you are done, click “End track” to end your track.
10. There should now be an extra pop-up window which contains the tracking data. Click it, then press ctrl + a (cmd + a for Mac) to select all the data. Press ctrl + c (cmd + c for Mac) and paste the tracking data into a spreadsheet software.
11. Repeat steps 1–10 for each zebrafish touch response video.
